# Use of CAD/CAM in Esthetic Restoration of Badly Decayed Tooth

**DOI:** 10.1155/2012/608232

**Published:** 2012-12-05

**Authors:** Satheesh B. Haralur, Ali Hassan Al-Faifi

**Affiliations:** Department of Prosthodontics, College of Dentistry, King Khalid University, P.O. Box 3263, Abha 61471, Saudi Arabia

## Abstract

Proper, intelligent use of materials and technology should be utilized for the benefit of the patient. This case report presents a patient with a badly broken premolar tooth demanding high esthetic all-ceramic restoration. Following multiple procedure tooth was restored with zirconia all ceramic restoration. Multiple procedures to save a tooth are a really worthy, in the interest of patients. Use of zirconia and CAD-CAM will help in saving many compromised tooth even in high-stress regions. This provides the patient a chance to have restorations, which are biomechanically superior in addition to being pleasing esthetically.

## 1. Introduction

Primary goal of every dentist is to perpetually preserve what is remaining than the meticulous replacement of what is missing [1]. Preservation of natural teeth is of paramount importance for many reasons, including integrity of arch, masticatory efficiency, esthetics, and phonetics. Loss of natural tooth has a profound negative effect on an individual self esteem and social relations [2, 3]. So dentist is duty bound to make every effort to save a valuable tooth. Modern day refined foods and lifestyle have resulted in increased incidence of dental caries [4]. Modern society is also obsessed with youthful appearance [5], so in dentistry esthetic demand is on the constant rise. 

For any restoration to be categorized as successful, it should not only satisfy the biomechanical needs but also esthetic need of a patient. Nonmetallic restorative materials such as all ceramic restorations are widely used because of optimal aesthetics, and color stability. Unfortunately, all ceramic crowns in the past were restricted to anterior low masticatory stress areas. 

Computer aided design/computer aided machining (CAD/CAM) is used widely in manufacturing industry for faster and precise production of components. Even though CAD-CAM was introduced to dentistry in mid-1980, only recently it has gained its popularity and widely accepted as an important restorative alternative. CAD-CAM technology is successfully utilized in dentistry for the fabrication of inlays, onlays, crowns, bridges, and even custom-made post. This technology helps in manufacturing the restorations with high precision and accuracy. This results in better adaptation and esthetics of restorations. Invent of CAD-CAM restoration made highly esthetic all ceramic restorations possible in a high stressful posterior region.

Yttrium-stabilized zirconium dioxide coping instead of metal for all ceramic crowns in high-stress areas is used successfully in recent years. CAD-CAM is used for the fabrication of zirconia copings. This alternative provides the strong, better adapted, highly esthetic restoration for the patients.

## 2. Case Presentation

**Figure 1:**
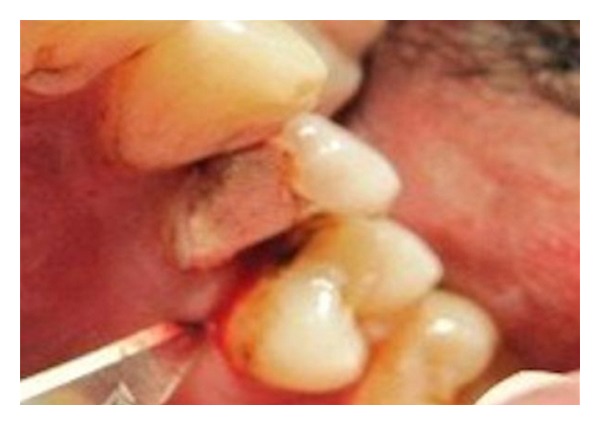
Badly decayed maxillary left premolar.

A 32-year-old male patient was presented to King Khalid University dental clinic with a badly decayed/broken upper left first premolar tooth for restoration (Figure 1). Though the tooth had fairly compromised restorative prognosis, patient was insisting on saving his tooth with esthetic restoration. Remaining anatomic crown structure was less than 30%. Tooth was evaluated for biological width, and intraoral radiographs were made to evaluate periodontal-endodontic condition of the teeth.

Intra-oral radiographs also confirmed chronic apical periodontitis. Only 0.5 mm of remaining tooth structure was present on the palatal surface of the tooth. On bone sounding evaluation, it was found that crown lengthening was needed to avoid biological width violation. As remaining tooth structure was less than 30%, tooth needed reinforcing with post and core restoration. Patient was treated sequentially with surgical crown lengthening procedure and root canal treatment. Since the patient was demanding the most esthetic restoration, prefabricated epoxy resin post was selected for reinforcing the weak crown structure. All ceramic zirconia crown was selected for the restoration of tooth to satisfy esthetic demand of the patient. 

**Figure 2:**
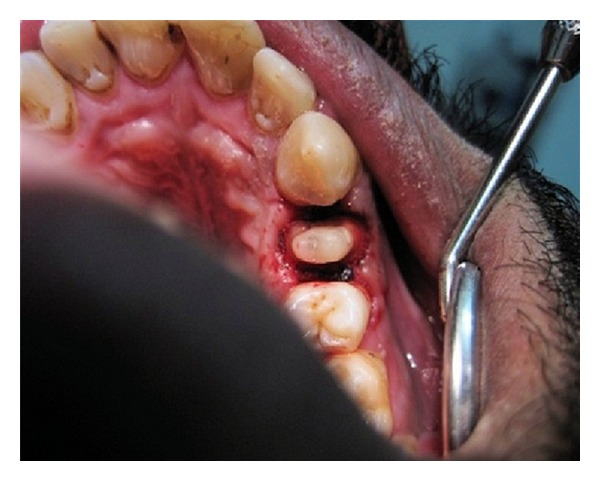
Photograph of prepared tooth after post and core and crown lengthening.

**Figure 3:**
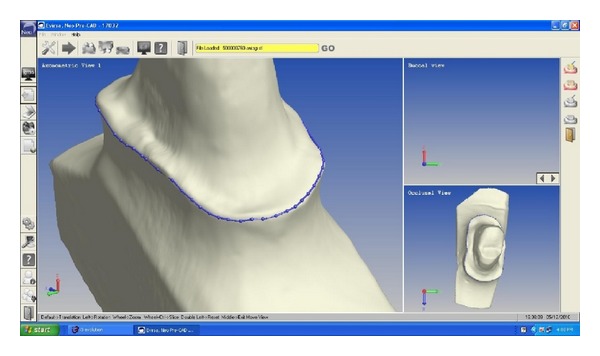
Die prototype in CAD-CAM.

**Figure 4:**
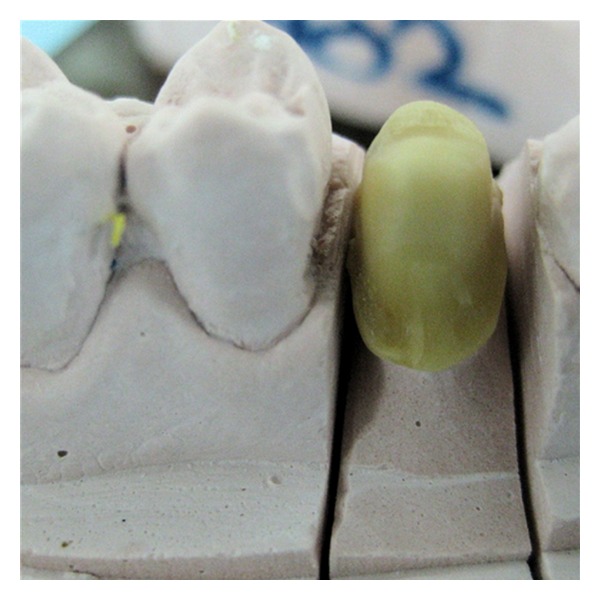
Milled zirconia coping.

**Figure 5:**
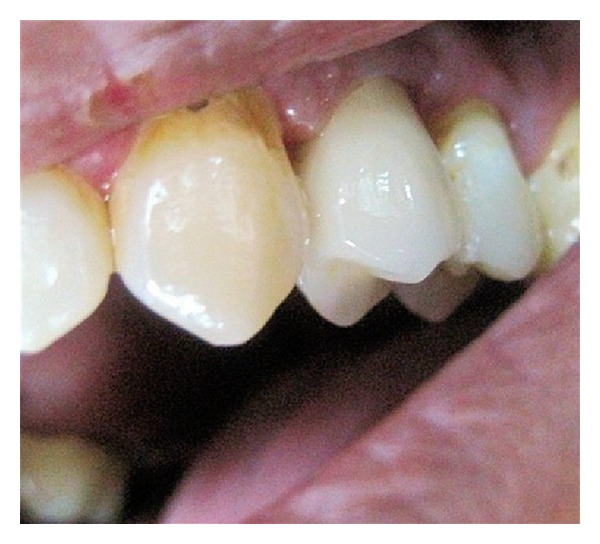
Final restoration in patient mouth.

The tooth was prepared with a deep chamfer finish line all around in addition to sufficient occlusal reduction (Figure 2). Impression was made with polyvinyl siloxane impression material. Zirconia coping was fabricated using CAD-CAM machine (Figures 3 and 4). After checking the fit of coping, it was veneered with vita ceramic porcelain. All ceramic zirconia crown was cemented with resin luting cement (Figure 5) and patient was given postcementation instruction.

## 3. Discussion

Non-metallic restorative materials such as all ceramic crowns are widely used because of optimal aesthetics, biocompatibility, color stability, high wear resistance, and low thermal conductivity [6]. Unfortunately, all ceramic crowns in the past were restricted to an anterior region with low masticatory stress. 

This case provided multiple challenges for all ceramic restorations; it had insufficient tooth structure, violation of biological width, presence of periapical pathology, and tooth in a high-stress area. Remaining tooth structure was barely 0.5 mm above free gingival margin. Tooth was evaluated for biologic width by bone sounding method. Biological width of minimum 2 mm along with 2 mm for ferrule preparation was calculated [7]. Surgical crown lengthening was performed to restore the biological width and enable circumferential ferrule preparation. Remaining tooth structure after preparation plays a major role in deciding the need of post and core [8]. Epoxy resin composite post was selected due to its esthetic nature and fewer incidences of root fracture [9].

Yttrium-stabilized zirconium dioxide coping instead of metal for all ceramic crowns in high-stress areas is successfully used in recent years [10, 11]. CAD-CAM is used for the fabrication of zirconia copings. Zirconia coping was selected instead of alumina coping to help in sustaining the expected high stresses in the premolar region. With careful evaluation of tooth and performing diligent and careful treatment procedure, dentist can save lots of hopeless teeth.

## 4. Conclusion

Every attempt should be made to save the natural teeth for the multiple benefits of a patient. Treatment plan should make provision to include the main requirement of the patient. Restoration without complete esthetic satisfaction of the patient is eventually a failure. CAD-CAM milled zirconia provides an opportunity for the dentist to provide a strong and esthetic restoration.
